# First-Principles Predictions and Synthesis of B_50_C_2_ by Chemical Vapor Deposition

**DOI:** 10.1038/s41598-020-61462-9

**Published:** 2020-03-10

**Authors:** Paul A. Baker, Wei-Chih Chen, Cheng-Chien Chen, Shane A. Catledge, Yogesh K. Vohra

**Affiliations:** Department of Physics, University of Alabama at Birmingham, Birmingham, Alabama 35294 USA

**Keywords:** Materials science, Mathematics and computing, Physics

## Abstract

Density functional theory predictions have been combined with the microwave-plasma chemical vapor deposition technique to explore metastable synthesis of boron-rich boron-carbide materials. A thin film synthesis of high-hardness (up to 37 GPa) B_50_C_2_ via chemical vapor deposition was achieved. Characterization of the experimental crystal structure matches well with a new theoretical model structure, with carbon atoms inserted into the boron icosahedra and 2b sites in a α-tetragonal B_52_ base structure. Previously reported metallic B_50_C_2_ structures with carbons inserted only into the 2b or 4c sites are found to be dynamically unstable. The newly predicted structure is insulating and dynamically stable, with a computed hardness value and electrical properties in excellent agreement with the experiment. The present study thus validates the density functional theory calculations of stable crystal structures in boron-rich boron-carbide system and provides a pathway for large-area synthesis of novel materials by the chemical vapor deposition method.

## Introduction

Boron-rich boron-carbide materials are of interest because of their thermal stability, high mechanical strength and their ability to function in extreme conditions of pressure, temperature, and corrosive environments. In general, materials for extreme environments typically contain at least one of the light elements C, N, O, and B, such as diamond and cubic boron nitride (c-BN). The short bond lengths of these light elements and the tendency to form directional covalent bonds make the structures difficult to compress or distort. Due to the high hardness of diamond, much research has been done on the synthesis of carbon-rich compounds with additions of boron and nitrogen, including the finding that the limit of solubility of boron in the diamond lattice is approximately 7.7 at%^1^. Boron-rich compounds are more difficult to categorize due to their tendency to form clusters of B_12_ icosahedra with interconnecting boron atoms in complex unit cells. Other elements typically insert into regions between icosahedra and can substantially change the mechanical and electronic properties of the materials. Boron-rich boron-carbide materials synthesis by chemical vapor deposition methods continues to be relatively unexplored and a challenging endeavor. In fact, even though boron was originally discovered in 1808, it was not produced in reasonably pure (99%) form until 1909. The pure phase is difficult to form due to impurities incorporating the lattice. The stable ambient pure form is still under contention, as there are α-tetragonal and β-rhombohedral phases that are thermodynamically stable under various conditions^2–6^. First-principles predictions have led to solutions for some of the pure and boron-rich phases^7–11^.

Researchers have found two difficulties when synthesizing boride compounds: achieving stoichiometric ratios uniformly throughout the material and keeping impurities from reacting with the boron during the synthesis process. Most of the novel high boron compounds are formed in high pressure high temperature (HPHT) cells, which produce very small volumes of material for analysis. Boron-rich boron-carbide in varying stoichiometric ratios has been made by HPHT methods^12^, but these methods are not scalable for producing large area coatings and can be difficult in controlling the impurities. On the other hand, microwave-plasma chemical vapor deposition (CVD) methods are better for controlling impurities in the material and can be used for large-area synthesis. The challenge is to find the correct set of conditions that are favorable for growth of the desired phase. In this study, we report the growth of high-hardness B_50_C_2_ thin films via CVD and experimentally characterize their properties by comparing to the predicted behaviors of a new theoretical stable and insulating B_50_C_2_ structure by first-principles calculations.

## Methods

### Film growth and characterization

The sample substrates were all sourced from a 550 nm silicon wafer (University Wafer ID: 1095) and cleaned in acetone, methanol, and DI water. The samples were all grown in a 2.45 GHz microwave-plasma chemical vapor deposition system (Wavemat MPDR 313EHP) using hydrogen as the carrier gas and diborane (90% H_2_, 10% B_2_H_6_, and ppm carbon) as the reactive gas. Low level of residual carbon has been found to appear consistently in the high-boron deposited films. The gas flow rates were: H_2_ = 500 Standard Cubic Centimeters per Minute SCCM, B_2_H_6_ (10%) =1 SCCM. Samples were grown at ~750 °C substrate temperature. The growth was performed at a pressure of 15 Torr using 1 kW of microwave power, and the deposition time per sample was 4 hours.

Characterization of the films was performed with X-ray photoelectron spectroscopy (XPS) using a Phi Electronics, Inc. Versaprobe 5000 equipped with a monochromatic Al X-ray source with a 100 um spot size at 25 W. The system has dual charge neutralization so no corrections were done to the peak positions. Survey scans were taken with 0.8 eV step size and a pass energy of 187.85 eV. High resolution scans were taken with 0.1 eV step size and a pass energy of 23.5 eV. Scanning Electron Microscopy (SEM) was performed with a FEI Quanta 650 FEG system. X-ray diffraction (XRD) analysis was performed using a Panalytical Empyrean system with a Cu anode (λ = 1.54187 Å) and Cu K-beta reducing incident optic with 1/8° divergence slit and 1/16° anti-scatter slits (quasi-parallel beam setup). On the diffracted optics side a parallel plate collimator (0.027° acceptance) with a proportional detector was used. The incident beam was fixed at 1° omega and the detector scanned from 10–100° 2θ. A Rietveld refinement was performed using the various structures and compared for weighted profile R-value (wRp). Hardness and Young’s modulus were measured using an MTS NanoIndenter XP having a Berkovich diamond tip with nominal radius of 50 nm. Calibration of the indenter area function before and after hardness measurements was tested on the fused silica standard (accepted Young’s modulus of 72 GPa) to confirm that the tip geometry did not change during testing of the B_50_C_2_ film. All indents, including those on silica, were made to a maximum depth of 150 nm. The measured Young’s modulus and hardness values were determined at maximum load. Young’s modulus of the silica before and after testing the CVD-grown B_50_C_2_ film was 72.4 ± 4.9 GPa and 72.8 ± 3.0 GPa, respectively. Therefore, the indenter tip area function was determined not to have changed significantly as a result of testing the film.

### First-principles predictions by density functional theory

The density functional theory (DFT)^13,14^ calculations are performed with VASP (the Vienna *ab initio* simulation package)^15,16^, in which a plane-wave basis set and pseudopotential method are adopted. In our calculations, we employ the projector augmented wave (PAW)^17,18^ method and Perdew-Burke-Ernzerhof (PBE-GGA)^19^ exchange correlation functional with a plane-wave kinetic cutoff energy of 600 eV. The Monkhorst-Pack k-point sampling of the Brillouin zone^20^ is chosen for a *Γ*-centered mesh with resolution = 0.02 × 2π/Å (6 × 6 × 10). The convergence criteria for self-consistent field and structure relaxation are set to 10^−8^ eV/unit cell and 10^−5^ eV/Å, respectively. We have made convergence tests with respect to k-points and cutoff energy. With a 6 × 6 × 10 k-grid and 600 eV cutoff energy, our calculations are able to achieve a total energy difference within 1 meV/atom. For electronic density of states, we adopt the tetrahedron method with a k-point sampling resolution = 0.01 × 2π/Å (12×12×20). In the phonon calculations, the interatomic force constants are obtained by density functional perturbation theory implemented in VASP, and the vibrational properties are computed by PHONOPY^21^. The magnitude of the displacement (*d*) in our B_50_C_2_ phonon calculations is 0.01 Å. To justify this value, we have first computed the phonon dispersions of α-Boron and cubic-diamond with *d* = 0.01 Å, and obtained good agreements with those in the literature. Moreover, for B_50_C_2_, we have further tested *d* = 0.008 Å and 0.012 Å in the phonon calculations, and the results are basically identical to that with *d* = 0.01 Å. Therefore, using *d* = 0.01 Å is appropriate in our study. The theoretical XRD patterns and structural visualization are plotted by the VESTA software^22^. In our calculations, we started with the tetragonal B_52_ structure reported in ref. ^11^, and then created a series of B_50_C_2_ structures by replacing 2 borons with 2 carbons. We have tried to put the 4 interstitial atoms in different symmetry sites as initial structures, and then fully relaxed the lattices and atomic positions before any further phonon and electronic structure calculations. Each B_50_C_2_ structure contains a unit cell of 52 atoms with a volume ~385 Å^3^. The lattice parameters for different B_50_C_2_ structures under study are summarized in Table 1.

**Table 1:** Comparison between the theoretically predicted and the experimental values of B_50_C_2_ lattice parameters and electrical properties. The structure in the first column was proven to be dynamically unstable.

Lattice Parameters	B_50_C_2_ (Theory-present work; structure from ref. ^11^) *Conductor*	B_50_C_2_ (Theory- present work) *Insulator*	B_50_C_2_ (Experiment – present work) *Insulator ρ ~MΩ-cm*	B_50_C_1.9_ α-tetragonal (ref. ^2^)
a	8.652 Å	8.724 Å	8.787 Å	8.753 Å
b	8.776 Å	8.784 Å	8.739 Å	8.753 Å
c	5.076 Å	5.013 Å	5.007 Å	5.093 Å
α	90°	89.77°	89.83°	90°
β	90°	89.70°	89.81°	90°
γ	90°	89.68°	89.64°	90°

The second and third columns show good agreement between theory and experiment.

## Results

### Characterization of films via XRD, XPS, raman, and nanoindentation

**Figure 1:**
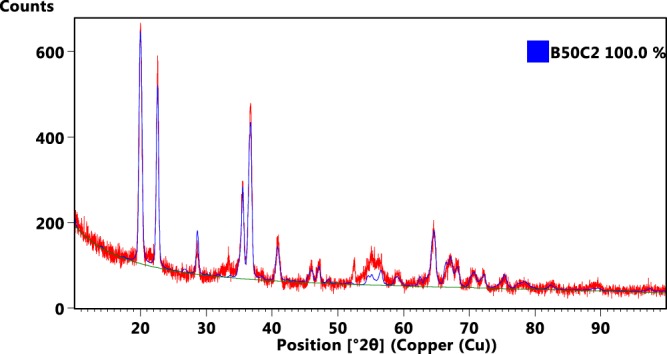
(Upper) Glancing angle x-ray diffraction of B_50_C_2_ grown at 750 °C on silicon substrate. The X-ray wavelength corresponds to Cu K-α emission (λ = 1.54187 Å). The red curve is the raw data and the blue curve is the Rietveld refinement with lattice parameters listed in Table 1.

Samples grown at 750 °C were found to contain only the B_50_C_2_ crystalline phase. The elemental analysis was confirmed by XPS and the phase determination was made using XRD analysis. No graphitic carbon or B_4_C were present as determined by XRD data (see Fig. 1). A Rietveld refinement was performed for B_50_C_2_ phase along with our newly-proposed insulating B_50_C_2_ phase. The most stable α-tetragonal B_52_ consists of four tetrahedrally coordinated B_12_ icosahedra, with four additional boron atoms occupying the interstitial 2b (0, 0, 0.5) and 4c (0, 0.5, 0) Wyckoff sites of the P4_2_/nnm space group^11^. A B_50_C_2_ structure can be formed by replacing boron with carbon in the B_52_ structure, or by inserting carbon into different interstitial sites. For example, previously reported B_50_C_2_ structures have carbons and interstitial boron inserted into the 8 h (0, 0.5, *z*), 8i (*x*, 0.5, 0.5), or 4c sites^11^. These reported B_50_C_2_ structures are metallic and found to be dynamically unstable in our phonon calculations. In contrast, the new B_50_C_2_ stoichiometric structure proposed here has carbon atoms inserted in both the B_12_ icosahedron and the 2b sites, and it is found to be dynamically stable and insulating (consistent with our measured resistivity value of deposited films to be ~MΩ-cm). Since the CVD process is at low pressure and the film grows by addition of atoms or molecules to the surface, reordering of the structure is not likely to happen. Thus the addition of carbon atoms within the boron icosahedra is likely to occur. Table 1 shows the lattice parameters of these structures.

The crystallographic determination was made using XRD. The graph of the XRD scan and the calculated fit to the data are shown in Fig. 1. The Panalytical program was used to fit the peaks and a search of the database for compounds with only C, N, O, and B as possible elements yielded the phases B_50_C_2_ and pure Boron (B_52_) as the most likely candidates. The new B_50_C_2_ phase was also added to the database by creating a line pattern in the software. A Rietveld fit was performed using each of these phases and the best fit to the experimental data was the new B_50_C_2_ phase with a weighted profile R-value (wRp) of 15 and the fit is shown in Fig. 1.

**Figure 2:**
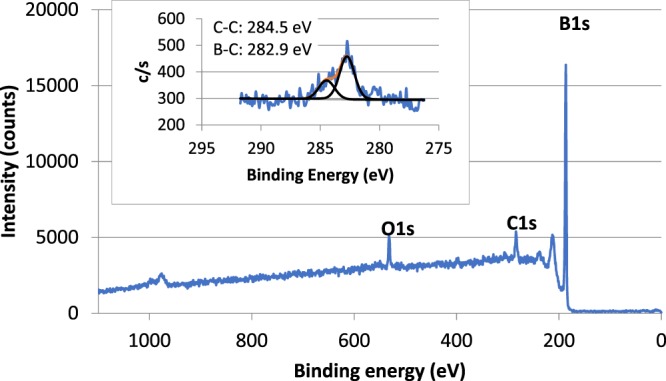
X-ray photoelectron spectroscopy scans of the B_50_C_2_ film with an elemental composition of 89.3% B, 7.8% C, and 3.0% O. The inset graph shows the high resolution scan of C1s showing a mixture of C-C (adventitious carbon) and B-C bonding (B_50_C_2_).

XPS of a B_50_C_2_ sample showed that the surface is composed of 89.3% B, 7.8% C, and 3.0% O (rel. at %) with no other elements present, as shown in Fig. 2. A small amount of surface contamination due to adventitious carbon is generally present in samples which have been exposed to air. Our high resolution scans in Fig. 2 insert shows that 40% of the carbon is C-C bonded (binding energy of 284.5 eV) and the remaining 60% is B-C bonded (binding energy of 282.9 eV). Using this information our XPS measured carbon content in the B-C bonded sample is 4.7%. The stoichiometric ratio for B_50_C_2_ should be 96% B: 4% C, and is close to our XPS measured value of 4.7%.

**Figure 3:**
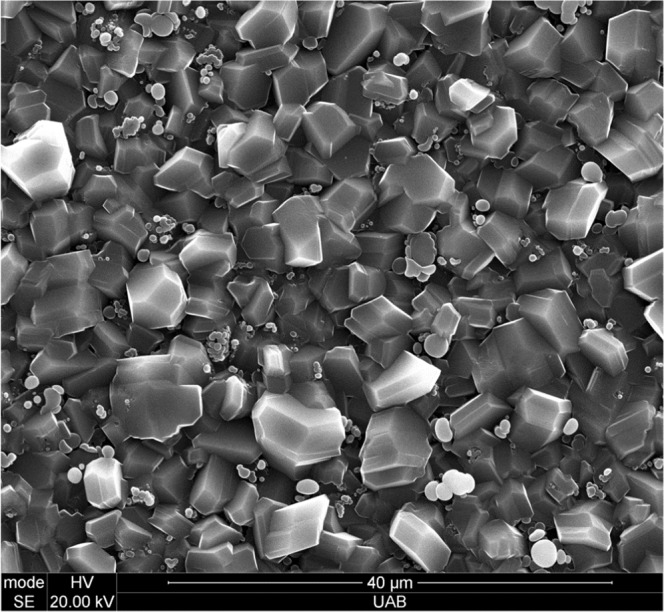
Scanning Electron Microscope (SEM) image of B_50_C_2_ crystallites grown on a silicon substrate. The scale bar at the bottom is 40 microns in length.

SEM imaging of the surface showed well-formed crystallites between 5 and 10 micrometers in size, as can be seen in Fig. 3. There does not appear to be any preferred orientation for the crystal growth. A large number of crystals appear to be twinned and many have stacking faults. These are likely due to the rapid growth (~2 μm/hour) of the crystals from the vapor phase.

Nanoindentation measurements were taken to a depth of 150 nm at several locations (N = 17 indents) on the film. The average hardness of all indents is 14 GPa, however, well faceted surfaces yield hardness as high as 37 GPa. The large spread in these hardness data can be attributed to the surface roughness causing the tip to make less than ideal initial surface contact during the load segment. It is interesting to note that the indent with the highest Young’s modulus Y did not have the highest hardness H (Y = 500 GPa and H = 25 GPa, respectively). This is not atypical of nanocomposite materials which have been reported with a non-linear modulus vs. hardness trend, and may be related to grain boundary deformation mechanisms^23–25^.

**Figure 4:**
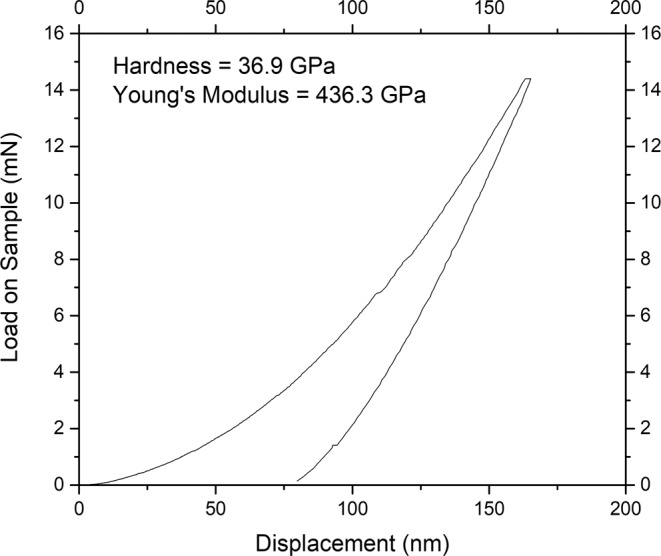
Nanoindentation load-displacement curve from one location on the B_50_C_2_ film to a depth of 150 nm. The extracted values of nanoindentation hardness and Young’s modulus are also indicated.

Figure 4 shows the load-displacement data for the 37 GPa indent, also labeled with measured Young’s modulus of 436 GPa. Although the indent depth was set to 150 nm, the maximum depth can be seen to be about 15 nm higher than this. This is due to a small correction made to the surface find segment to ensure a sharp increase in stiffness (corresponding to a displacement set at 0 nm) when indenter contact was made. The nanoindentation hardness of B_50_C_2_ is 37% of the hardness value for cubic diamond and Young’s modulus is about 41% of the value for the cubic diamond.

**Figure 5:**
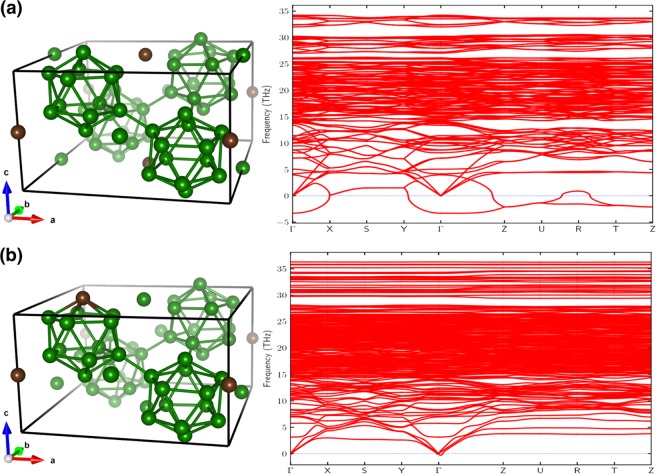
Crystal structures and phonon spectra for B_50_C_2_ with (**a**) carbon atoms inserted only into the 2b sites of a base B_52_ α-tetragonal phase, and (**b**) carbon atoms inserted into both the B_12_ icosahedron and 2b sites. The negative phonon modes for the structure shown in (**a**) indicates its dynamical instability.

Figure 5 shows the DFT calculations of phonon spectra for two different B_50_C_2_ structures. The structure in Fig. 5(a) has been considered previously in the literature^11^. This B_50_C_2_ structure is metallic, and it can be obtained by inserting carbon atoms into the 2b sites in a B_52_ α-tetragonal base structure (or equivalently into two of the 4c sites after a lattice translation and mirror reflection). However, our phonon calculation [Fig. 5(a), right panel] indicates negative phonon modes, showing that the corresponding crystal structure is actually dynamically unstable. By examining the phonon eigenvector at the Γ point, we found that the unstable mode has a predominant contribution from the two interstitial borons. This result suggests that relaxing the boron positions away from the 4c sites could potentially lead to a lower energy structure. On the contrary, in the structure shown in Fig. 5(b), the corresponding phonon modes are all non-negative, and thereby the newly predicted B_50_C_2_ structure is dynamically stable. This new B_50_C_2_ structure has one carbon replacing boron in the B_12_ icosahedron^26^ and the other carbon inserted in one of the 2b sites (or equivalently one of the 4c sites after a lattice translation and mirror reflection). We note that in the new B_50_C_2_ structure, one interstitial boron atom relaxes to a Wyckoff position (0.488, 0.915, 0.575), and the other one to a site near 8 h (0, 0.5, *z*) with *z* ~0.189. This agrees with previous finding that the 8 h site is occupied in B_50_C_2_^2^, but not in B_52_^3^. We also note that since B_50_C_2_ is a 3D material with strong covalent bonding, we do not expect much difference in the structure relaxation by employing a DFT-D3 correction^27^, which is relevant for layered materials or large molecules with van der Waals interaction. For the tetragonal B_50_C_2_, for example, we have checked that the lattice parameter *a* shows a difference of only ~0.023 Å (~0.26%) by applying a DFT-D3 correction, which is consistent with our expectation that such effect will not cause an appreciable difference in our study.

**Figure 6:**
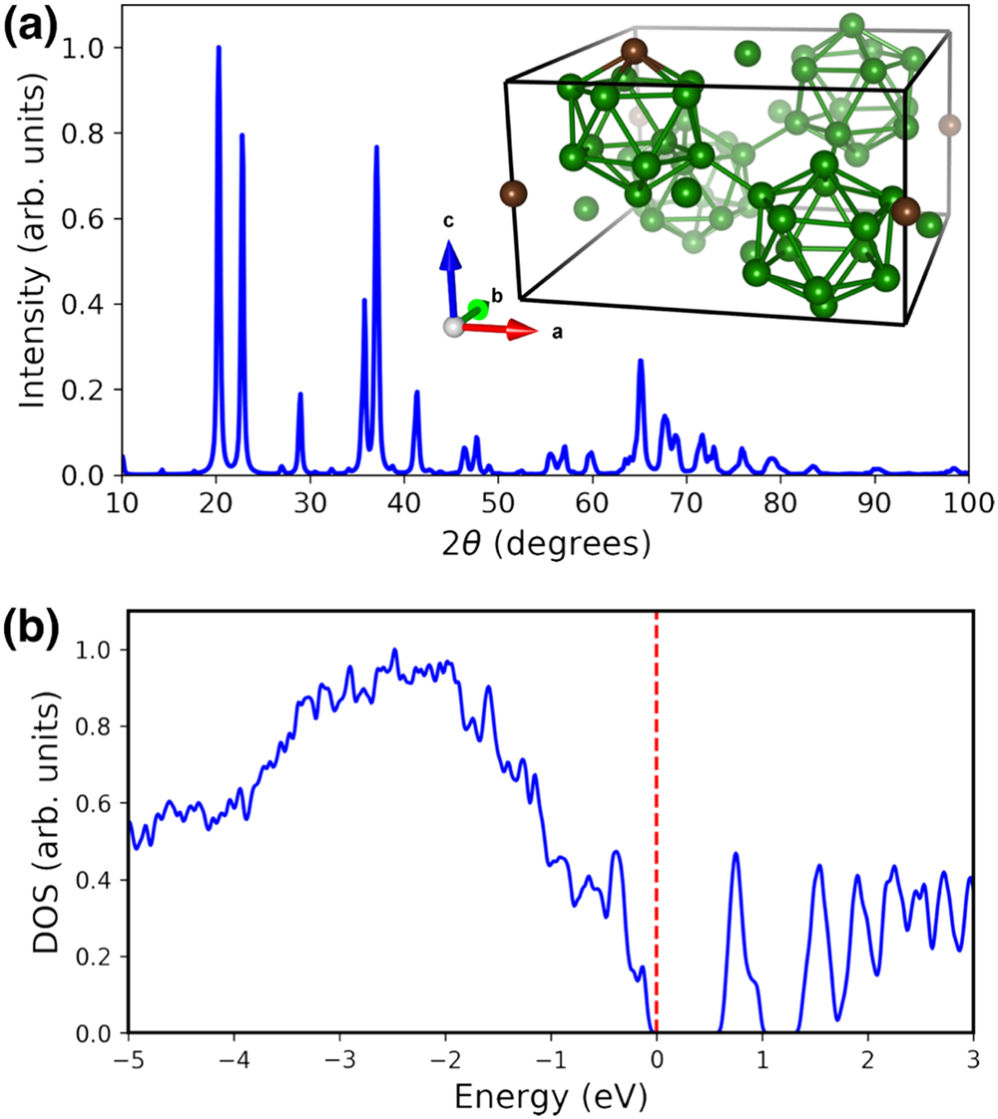
(**a**) Cu K-α X-ray diffraction pattern computed for the new theoretical B_50_C_2_ structure shown in the inset. (**b**) Electronic density of states (DOS) for the new structure showing that the Fermi level (indicated by the vertical dashed line) lies at the top of the valence band, which is well separated from the conduction band by a finite energy gap ~0.7 eV. The electronically insulating behavior also makes the structure dynamically stable.

The Cu K-α XRD pattern computed for the new theoretically stable B_50_C_2_ structure is shown in Fig. 6(a), which also matches well the experimental XRD pattern in Fig. 1, where the computed phase is fit to the experimental data. Moreover, it is noted the previously reported (unstable) B_50_C_2_ structure [Fig. 5(a)] is metallic, while the stable B_52_ and B_50_N_2_ structures are both insulating^7^. Indeed, a corresponding calculation of the electronic density of states (DOS) shown in Fig. 6(b) demonstrates that the new theoretically stable B_50_C_2_ is also insulating, which potentially helps its structure stability. We note that the carbon position plays the most import role in changing the electrical property. In particular, when a carbon atom is placed within one of the four B_12_ icosahedra, the Fermi level will move to the valence band top, and a gap ~ 0.7 eV will appear. This behavior also occurs in other superhard B-C system. For example, it is known that B_12_C_3_ has two different structures B_12_(CCC) and B_11_C(CBC). The lower symmetry structure B_11_C(CBC) consists of B_11_C icosahedra and C-B-C chains, and it also has a lower total energy with a bandgap ~ 1.4 eV larger than that of B_12_(CCC), which has carbon atoms placed outside the B_12_ icosahedron. Our results on B_50_C_2_ thereby agree with previous findings on B_12_C_3_^28^. Finally, additional DFT calculation using Chen’s model^29^ finds a Vickers hardness around 36 GPa for our newly reported B_50_C_2_ structure, also in excellent agreement with the hardness measurement.

## Conclusions

We have produced a boron-rich boron-carbide phase B_50_C_2_ by microwave-plasma chemical vapor deposition over an area of 25 mm^2^, and have shown that it more closely resembles a crystal structure which includes carbon atoms inserted in the icosahedra of boron as well as in sites between icosahedra. This is opposed to previously proposed structures that are found to be dynamically unstable in our phonon calculations. The new phase has a high hardness value in excellent agreement with the experimentally measured value of 37 GPa. The new phase is predicted to be insulating by electronic band structure calculations in agreement with our measured resistivity value of MΩ-cm. Our present studies thus provide validation of the density functional theory in predicting stable crystal structure and providing a metastable synthesis pathway for boron-rich boron-carbide materials for applications under extreme conditions of pressure, temperature, and corrosive environments.
